# Association of Bolus Feeding With Splanchnic and Cerebral Oxygen Utilization Efficiency Among Premature Infants With Anemia and After Blood Transfusion

**DOI:** 10.1001/jamanetworkopen.2020.0149

**Published:** 2020-02-28

**Authors:** Kiran Kumar Balegar V, Madhuka Jayawardhana, Andrew J. Martin, Philip de Chazal, Ralph K. H. Nanan

**Affiliations:** 1 Department of Neonatology, Sydney Medical School Nepean, Nepean Hospital, The University of Sydney, Kingswood, Australia; 2 School of Biomedical Engineering, The University of Sydney, Sydney, Australia; 3 NHMRC Clinical Trials Centre, The University of Sydney, Camperdown, Australia; 4 The Charles Perkins Center, The University of Sydney, Sydney, Australia

## Abstract

**Importance:**

The pathogenesis of transfusion-associated necrotizing enterocolitis remains elusive. Splanchnic hypoperfusion associated with packed red blood cell transfusion (PRBCT) and feeding has been implicated, but studies of splanchnic tissue oxygenation with respect to feeding plus PRBCT are lacking.

**Objective:**

To investigate the oxygen utilization efficiency of preterm gut and brain challenged with bolus feeding during anemia and after transfusion using near-infrared spectroscopy.

**Design, Setting, and Participants:**

This prospective cohort study conducted from September 1, 2014, to November 30, 2016, at a tertiary neonatal intensive care unit included 25 hemodynamically stable infants with gestational age less than 32 weeks, birth weight less than 1500 g, and postmenstrual age younger than 37 weeks. Data analysis was performed from August 1, 2017, to October 31, 2018.

**Exposures:**

Infants received PRBCT (15 mL/kg for 4 hours) and at least 120 mL/kg daily of second hourly bolus feedings.

**Main Outcomes and Measures:**

Splanchnic fractional tissue oxygen extraction (FTOEs) and cerebral fractional tissue oxygen extraction (FTOEc) measures were made during 75-minute feeding cycles that comprised a 15-minute preprandial feeding phase (FP0) and 4 contiguous 15-minute postprandial feeding phases (FP1, FP2, FP3, and FP4; each 15 minutes long). The intraindividual comparisons of feeding-related changes were evaluated during the pretransfusion epoch (TE0: 4 hours before onset of transfusion) and 3 TEs after transfusion (TE1: first 8 hours after PRBCT completion; TE2: 9-16 hours after PRBCT completion; and TE3: 17-24 hours after PRBCT completion).

**Results:**

Of 25 enrolled infants (13 [52%] female; median birth weight, 949 g [interquartile range {IQR}, 780-1100 g]; median gestational age, 26.9 weeks [IQR, 25.9-28.6 weeks]; median enrollment weight, 1670 g [IQR, 1357-1937 g]; and median postmenstrual age, 34 weeks [IQR, 32.9-35 weeks]), 1 infant was excluded because of corrupted near-infrared spectroscopy data. No overall association was found between FTOEs and FPs in a multivariable repeated-measures model that accounted for transfusion epochs (primary analysis approach) (FP0: mean estimate, 11.64; 95% CI, 9.55-13.73; FP1: mean estimate, 12.02; 95% CI, 9.92-14.11; FP2: mean estimate, 12.77; 95% CI, 10.68-14.87; FP3: mean estimate, 12.54; 95% CI, 10.45-14.64; FP4: mean estimate, 12.98; 95% CI, 10.89-15.08; *P* = .16 for the FP association). However exploratory analyses of postprandial changes in FTOEs undertaken for each transfusion epoch separately found evidence of increased postprandial FTOEs during TE1 (mean [SD] FTOEs, 10.55 [5.5] at FP0 vs 13.21 [5.96] at FP4, *P* = .046). The primary and exploratory analyses found no association between FTOEc and feeding phases, suggesting that cerebral oxygenation may be protected.

**Conclusions and Relevance:**

The findings suggest that enteral feeding may be associated with gut ischemia and potentially transfusion-associated necrotizing enterocolitis. The postprandial changes in FTOEs appear to warrant further investigation in larger randomized studies.

## Key Points

**Question:**

Is there an association between feeding and splanchnic and/or cerebral oxygen utilization efficiency during anemia and/or after packed red blood cell transfusion?

**Findings:**

In this cohort study of 25 hemodynamically stable, bolus-fed preterm infants who received packed red blood cell transfusions, splanchnic fractional tissue oxygen extraction was adversely associated with feedings in the immediate (first 8 hours) posttransfusion period. Cerebral fractional tissue oxygen extraction was protected during the anemia and posttransfusion periods.

**Meaning:**

The findings suggest that enteral feeding may be associated with gut ischemia and potentially transfusion-associated necrotizing enterocolitis and reiterate cerebral autoregulation in this context.

## Teaser

This cohort study assesses the oxygen use efficiency of the gut and brain challenged with bolus feeding during anemia and after blood transfusion in preterm infants.

## Introduction

Several studies^1,2,3,4,5,6,7^ suggest a temporal association between packed red blood cell transfusion (PRBCT) in the 48 to 72 hours preceding PRBCT and the development of necrotizing enterocolitis (NEC) in low-birth-weight infants. Compared with classic NEC, transfusion-associated NEC (TANEC) cases are more severe,^2^ with higher rates of surgical intervention^6^ and mortality.^1^ In general, TANEC has delayed postnatal age at onset, and infants tend to be of lower gestational age and birth weight, to be sicker on the first days of life, and to have higher odds of having patent ductus arteriosus (PDA).^1,2,3,5,6,8,9,10,11,12,13^ Cases of TANEC are reported to be associated with transfusion of red blood cells stored for a longer period and transfusion given for significant anemia.^11^ Although the pathogenesis of TANEC is unclear, a proposed hypothesis includes transfusion-induced splanchnic hypoperfusion, further exacerbated by feeding^5,14,15^ in the context of anemia,^15,16,17^ and vulnerable splanchnic microcirculation in premature infants.^18^ Some clinical studies^2,6,7^ have found variable results regarding the role of feeding in infants with TANEC, with some showing no benefit of withholding feeding from infants during the transfusion period, whereas others^3,4,5,12^ suggest an association between feeding and TANEC. As a result, feeding practices during the peritransfusion period are variable in different neonatal intensive care units.^19,20^ Physiologic studies in animals^16,21^ and preterm newborns^15,22^ on the association of anemia and transfusion with postprandial mesenteric blood flow and/or tissue oxygen levels are limited. Prospective physiologic data using near-infrared spectroscopy (NIRS) to evaluate the interplay between splanchnic oxygen delivery and consumption are necessary to better understand the oxygen utilization efficiency of preterm gut challenged with bolus feeding during anemia and after transfusion.

Although cerebral autoregulation and its mechanism in preterm infants have been extensively investigated,^23^ there is a paucity of studies evaluating the dynamic equilibrium between cerebral oxygenation consumption and supply during feeding and transfusion, and the available studies^17,24^ have produced variable results.

The current study was conducted to evaluate the association of bolus enteral feeding on splanchnic and cerebral tissue bed oxygenation in anemia and after PRBCT using NIRS and to assess the duration of any association after transfusion. We hypothesized that splanchnic tissue oxygenation would be reduced after feedings in infants with anemic status and would be worse rather than improved after PRBCT. We also hypothesized that cerebral oxygenation would be unaffected because of its autoregulatory capacity.

## Methods

This prospective cohort study was conducted in the tertiary neonatal intensive care unit at Nepean Hospital, Sydney, Australia, from September 1, 2014, to November 30, 2016. Data analysis was performed from August 1, 2017, to October 31, 2018. Eligibility criteria were as follows: gestational age less than 32 weeks, birth weight less than 1500 g, postmenstrual age younger than 37 weeks, tolerating total daily feeding volume at least 120 mL/kg, and hemodynamically stable and receiving elective PRBCT to treat anemia of prematurity. Infants with NEC (current or previous); feeding intolerance (defined as the treating clinical team’s decision to withhold feedings or withhold grading up of feedings for at least 12 hours); sepsis (defined as the treating clinical team’s decision to commence antibiotic treatment); PRBCT in the previous 72 hours; PDA or its treatment with ibuprofen, indomethacin, or surgery in the previous 72 hours; and/or congenital gastrointestinal, complex cardiac, or lethal anomalies were excluded. An infant was enrolled only once even when receiving multiple transfusions. For pragmatic reasons, enrollment occurred only when PRBCT was initiated during normal business hours (8 am to 5 pm weekdays). A pragmatic sample size of 25 was selected to facilitate reasonable enrollment based on the number of potentially eligible infants typically admitted to the neonatal intensive care unit and the rate of transfusion. Consecutive infants who satisfied these criteria were enrolled after a written informed parental consent was obtained by the chief investigator (K.K.B.V.) or one of the designated officers. All data were deidentified. The study protocol, including parental consent, was approved by the Nepean Blue Mountain Local Health Committee. This study followed the Strengthening the Reporting of Observational Studies in Epidemiology (STROBE) reporting guideline.

### Transfusion and Bolus Feedings

Once a clinical decision was made for transfusion, the study period extended from 4 hours before the PRBCT until 24 hours after its completion. The decision to administer PRBCT was made by the attending neonatal team composed of neonatologists (including one of us [K.K.B.V.]), neonatal trainees, and neonatal nurses independent of the study. No formal protocol existed regarding the hemoglobin threshold at which infants received a PRBCT. The decision to transfuse was made based on a combination of clinical factors in addition to low hemoglobin level (eg, respiratory support, increasing desaturations, poor growth, gestation, and reticulocyte response). Infants received PRBCT at 15 mL/kg for 4 hours without furosemide. Type (breast milk vs formula) and volume of feeding were determined by the clinical team independent of the study but remained the same during the entire study period. Only infants who tolerated a reasonable feeding volume (defined as ≥120 mL/kg daily) were enrolled because of concerns that feeding intolerance attributable to other reasons (eg, premature sluggish gut or NEC) may confound splanchnic oxygenation independent of feeding. For standardization across enrolled infants during the study period, the second hourly aliquots of total daily feeding volume were given as bolus via gastric tube at a constant rate using a syringe pump. The rate was chosen as 120 mL per hour based on the mean rate of gavage feeding in a cohort of 30 preterm infants. A standardized rate of bolus feedings was given to avoid potential feeding intolerance (such as vomiting or regurgitation) because of variation in the rate of the bolus by other methods, such as gavage feedings or hand push.

### NIRS Monitoring and FTOE Calculation

The NIRS measures oxygen saturation in the tissue bed approximately 1 to 2 cm beneath the sensor and displays it as regional tissue oxygen saturation (Sto_2_) on a scale from 15% to 95%.^25,26,27,28,29,30,31,32^ Through the simultaneous monitoring of arterial oxygen saturation as measured by pulse oximetry (Spo_2_) and StO_2_, FTOE was calculated as follows: FTOE = ([SpO_2_ – StO_2_] × 100/[SpO_2_]). The FTOE indicates the efficiency of oxygen utilization and is determined by oxygen consumption in the context of prevailing oxygen delivery (FTOE = [oxygen consumption/oxygen delivery]). FTOEc has been studied in preterm infants as a surrogate measure of the adequacy of consumption vs delivery. An increase in FTOE indicates inefficient tissue oxygen utilization owing to consumption being out of proportion to delivery.^33,34^ Similarly, a decrease in FTOE indicates efficient tissue oxygen utilization with delivery being adequate for tissue needs. A change in FTOE rather than absolute values is meaningful.

During the entire study period, a continuous prospective evaluation of StO_2_ was performed using a 4-wavelength NIRS cerebral and splanchnic tissue monitor (FORE-SIGHT absolute cerebral oximeter, CAS Medical Systems Inc) placed on the lower quadrant of the abdomen just below the umbilicus^28,35^ to obtain splanchnic StO_2_ and a second neonatal sensor placed over the temporal region of the head to obtain cerebral StO_2_. SpO_2_ was monitored using the Masimo Radical-7 monitor (Radical-7 Pulse CO-Oximeter, Masimo Corp) for concurrent measurement of arterial oxygen saturation. For infants who needed supplemental oxygen, fraction of inspired oxygen was adjusted to a target Spo_2_ of 91% to 95%, as per the unit protocol. Skin integrity was closely monitored by lifting the sensors and inspecting the skin every 6 hours during care tasks (eg, handling of neonates for a diaper change, eye care, and change of posture). The start and end times of the study, as well as various events (transfusion, feedings, and care tasks), were electronically annotated in real time. As a backup, these events were also recorded on a hard copy datasheet. Other demographic information (Table 1) was also collected.

**Table 1.** Main Clinical Characteristics of the Study Participants^a^

Characteristic	Finding (n = 25)
Baseline characteristics	
Gestational age, median (IQR), wk	26.9 (25.9-28.6)
Birth weight, median (IQR), g	949 (780-1100)
Female	13 (52)
Restricted umbilical arterial flows on antenatal Doppler studies	2 (8)
Small for gestational age	3 (12)
Enrollment characteristics	
Postmenstrual age, median (IQR), wk	34 (32.9-35)
Postnatal age, median (IQR), d	42 (27-59)
Weight, median (IQR), g	1670 (1357-1937)
Breathing support	
None	5 (20)
Continuous positive airway pressure	10 (40)
High-flow nasal cannula	8 (32)
Low-flow oxygen	2 (8)
Daily feed intake, median (IQR), mL/kg	160 (150-160)
Nature of feedings	
Breast milk without fortification	1 (4)
Breast milk with fortification	15 (60)
Preterm formula milk	9 (36)
Caffeine	20 (80)
Patent ductus arteriosus	0
Transfusion characteristics, median (IQR)	
Pretransfusion hemoglobin, g/dL	9.3 (8.6-10.3)
Packed red blood cells hemoglobin, g/dL	19.7 (18.8-20.0)
Age of packed red cell transfusion, median (IQR), d	9 (4-23)
Necrotizing enterocolitis within 72 h of the study period	0
Feed intolerance within 72 h of the study period	0

Abbreviation: IQR, interquartile range.

SI conversion factors: To convert hemoglobin to grams per liter, multiply by 10.

^a^ Data are presented as number (percentage) of study participants unless otherwise indicated.

### Data Processing

The StO_2_ and SpO_2_ data were downloaded as an analog output at a sampling rate of 1000 Hz and aligned along the time axis in LabChart reader format (.adicht files) using a PowerLab data acquisition system^36^ (ADInstruments). The .adicht files were converted into .mat file format using a simple Python script (Python, version 3.7.3^37^) and resampled at 1 Hz for faster processing. Data that could not be physiologically explained (eg, absence of variability^35^ or a 30% step change in StO_2_ between 2 subsequent data points for StO_2_^38^) were removed. Data during the care tasks were presumed to be artifactual for 2 reasons. First, NIRS sensors were lifted for inspection of underlying skin during this period. Second, infants underwent diaper change, oral care, change of position, and other events during this period that would have caused significant movement artifacts. The removed segments were replaced with the phrase “not a number,” which is recognized by Matlab,^39^ version 9.3 (The MathWorks Inc) and ignored for all subsequent processing.

**Figure 1.**
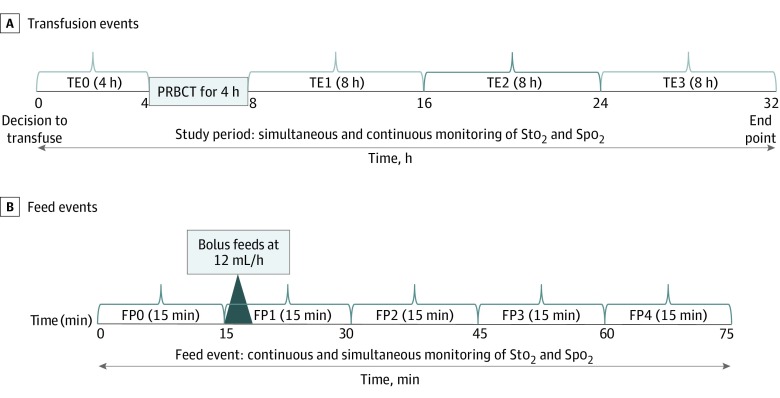
Outline of the Study Procedure and Feeding Analysis FP indicates feeding phase; PRBCT, packed red blood cell transfusion; Spo_2_, oxygen saturation as measured by pulse oximetry; Sto_2_, tissue oxygen saturation; and TE, transfusion epoch.

The 4-hour period before the beginning of a transfusion was designated as transfusion epoch (TE) 0. The 24-hour period after the completion of transfusion was divided into 3 epochs each of 8 hours’ duration (TE1: first 8 hours; TE2: 9-16 hours; and TE3: 17-24 hours) (Figure 1A). Because the feeding timings in relation to transfusion varied in different infants, a single feeding event was chosen for the analysis from each TE based on predefined criteria as the first feeding event during each TE that did not overlap with care tasks. No feeding analysis was performed during the intratransfusion period because most feeding events extended on either side of the intratransfusion period.

Each feeding event within each epoch was divided into a preprandial feeding phase (FP0: 15 minutes before the beginning of a feeding) and 4 postprandial feeding phases (FP1: 0-15 minutes after the beginning of the feeding; FP2: 16-30 minutes after the beginning of the feeding; FP3: 31-45 minutes after the beginning of the feeding; and FP4: 46-60 minutes after the beginning of the feeding) (Figure 1B). The predefined periods were chosen to capture tissue oxygenation changes at different time points after the commencement of a feeding because feeding is associated with a significant and progressive increase in superior mesenteric artery flow that commences by 15 minutes and peaks by 30 to 45 minutes after a feeding.^40,41,42^ Mean FTOEs and FTOEc were derived from the real-time StO_2_ and SpO_2_ data for each infant within FPs (FP0-FP4) and TEs (TE0-TE3).

### Statistical Analysis

We analyzed FTOEs and FTOEc using a mixed model for repeated measures (MMRM) with FPs (FP0-FP4) and TEs (TE0-TE3) fitted as factors. We tested whether the association between FTOE and FPs differed across TEs by fitting an interaction term to the MMRM. The primary MMRM modeling approach (with TE and FP fitted as factors) accommodated imbalance designs and included all 24 infants. In an exploratory set of analyses (number of infants given in Table 2), we applied an MMRM model to each transfusion epoch separately (ie, 4 models per FTOE measure) and performed a series of paired comparisons between FP1 to FP4 vs FP0. The Dunnett method was used to adjust for the multiplicity of *P* values from these paired comparisons. All *P* values were 2-sided, and *P* < .05 was considered statistically significant.

**Table 2.** Exploratory Analysis of FTOEs and FTOEc in Different FPs and TEs^a^

TE, FP	FTOEs				FTOEc			
	No.	Mean (SD), %	Estimated Difference From FP0, % (95% CI)	*P* Value	No.	Mean (SD), %	Estimated Difference From FP0, % (95% CI)	*P* Value
0								
0	24	12.06 (7.37)	NA	NA	24	26.23 (5.43)	NA	NA
1	24	11.68 (7.54)	−0.39 (−2.32 to 1.55)	.97	24	26.67 (5.57)	0.45 (−0.38 to 1.28)	.48
2	24	13.01 (6.91)	0.95 (−0.99 to 2.88)	.56	24	26.41 (5.62)	0.19 (−0.64 to 1.01)	.95
3	24	12.92 (6.70)	0.86 (−1.07 to 2.80)	.64	24	25.40 (5.84)	−0.82 (−1.65 to 0.01)	.052
4	24	13.59 (7.12)	1.53 (−0.40 to 3.46)	.16	24	25.58 (5.11)	−0.65 (−1.48 to 0.18)	.17
1								
0	22	10.55 (5.50)	NA	NA	22	20.45 (3.04)	NA	NA
1	22	11.73 (5.18)	1.18 (−1.45 to 3.81)	.63	22	20.97 (4.14)	0.52 (−0.63 to 1.66)	.62
2	22	12.57 (5.70)	2.03 (−0.60 to 4.65)	.18	22	20.80 (3.74)	0.35 (−0.80 to 1.49)	.86
3	22	12.80 (5.26)	2.26 (−0.37 to 4.88)	.11	22	20.27 (3.31)	−0.18 (−1.33 to 0.96)	.98
4	22	13.21 (5.96)	2.66 (0.04 to 5.29)	.046	22	20.05 (3.50)	−0.40 (−1.55 to 0.74)	.79
2								
0	23	11.16 (5.46)	NA	NA	23	21.46 (6.49)	NA	NA
1	22	12.17 (4.24)	0.89 (−1.21 to 2.99)	.67	22	21.49 (5.05)	−0.36 (−1.79 to 1.08)	.93
2	22	12.63 (4.54)	1.36 (−0.74 to 3.45)	.31	22	21.96 (4.50)	0.11 (−1.32 to 1.55)	>.99
3	22	11.87 (5.11)	0.60 (−1.50 to 2.69)	.89	22	21.41 (4.93)	−0.44 (−1.88 to 0.99)	.86
4	22	11.98 (4.94)	0.70 (−1.39 to 2.80)	.82	22	21.35 (4.63)	−0.50 (−1.93 to 0.94)	.80
3								
0	21	12.79 (7.06)	NA	NA	21	23.00 (5.78)	NA	NA
1	21	12.55 (6.95)	−0.24 (−2.72 to 2.23)	>.99	21	23.46 (6.00)	0.46 (−0.37 to 1.29)	.44
2	21	12.85 (7.89)	0.06 (−2.42 to 2.53)	>.99	21	23.22 (5.92)	0.22 (−0.61 to 1.05)	.91
3	21	12.53 (8.56)	−0.27 (−2.74 to 2.21)	>.99	21	22.69 (6.00)	−0.31 (−1.14 to 0.52)	.76
4	21	13.09 (7.19)	0.30 (−2.18 to 2.77)	.99	21	22.69 (5.69)	−0.31 (−1.14 to 0.52)	.76

Abbreviations: FP, feeding phase; FTOEc, cerebral fractional tissue oxygen extraction; FTOEs, splanchnic fractional tissue oxygen extraction; NA, not applicable; TE, transfusion epoch.

^a^ The Dunnett method was used to correct *P* values and 95% CIs for the 4 pairwise tests per TE.

## Results

### Demographic Features

**Figure 2.**
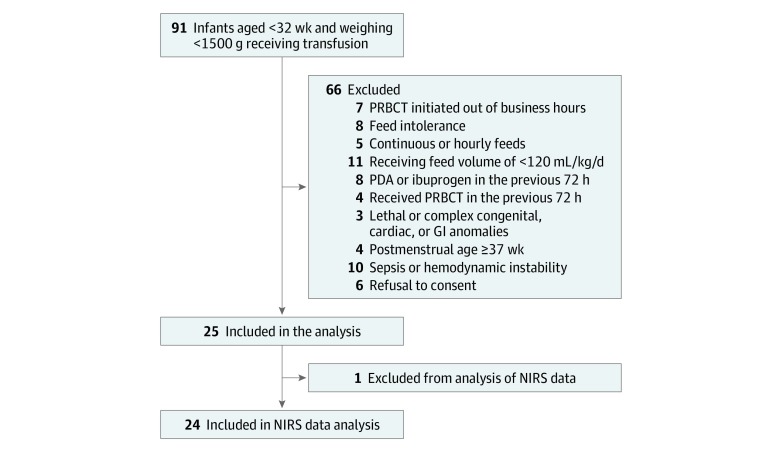
Flow Diagram of the Study Participants GI indicates gastrointestinal; NIRS near-infrared spectroscopy; PDA, patent ductus arteriosus; and PRBCT, packed red blood cell transfusion.

Of 25 enrolled infants (13 [52%] female; median birth weight, 949 g [interquartile range {IQR}, 780-1100 g]; median gestational age, 26.9 weeks [IQR, 25.9-28.6 weeks]; median enrollment weight, 1670 g [IQR, 1357-1937 g]; and median postmenstrual age, 34 weeks [32.9-35.0 weeks]), 1 infant was excluded because of corrupted NIRS data. Characteristics of potentially eligible, excluded, and analyzed infants are depicted in Figure 2. The characteristics of the study cohort are presented in Table 1. Two infants (8%) had restricted umbilical artery flows on antenatal Doppler studies, and 3 (12%) were small for gestational age. At enrollment, 5 infants (20%) were spontaneously breathing in room air without any respiratory support, 2 (8%) needed low-flow oxygen, 8 (32%) needed high-flow nasal cannula, and 10 (40%) received continuous positive airway pressure support. None had PDA; 20 (80%) received caffeine, and 9 (36%) received formula milk during the study period. The median daily feeding intake was 160 mL/kg daily (IQR, 150-160 mL/kg daily). The median pretransfusion hemoglobin level was 9.3 g/dL (IQR, 8.6-10.3 g/dL) (to convert to grams per liter, multiply by 10), and the age of transfused blood pack was 9 days (IQR, 4-23 days). None of the enrolled infants developed feeding intolerance or NEC within 72 hours after PRBCT.

### Regional Splanchnic Tissue Oxygenation

**Figure 3.**
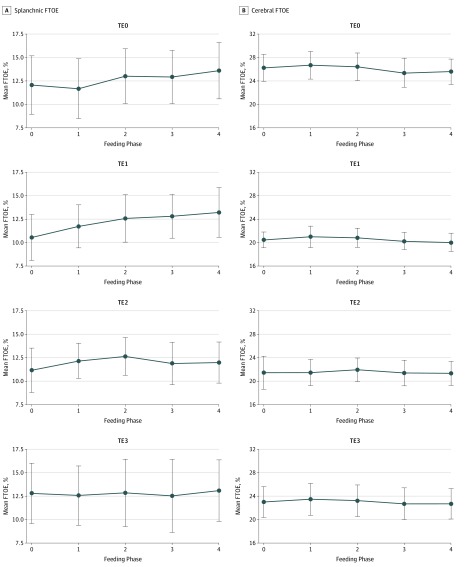
Feeding-Related Changes in Splanchnic Fractional Tissue Oxygen Extraction (FTOEs) and Cerebral FTOE During Different Transfusion Epochs (TEs) No feeding-associated changes were noted in cerebral FTOE during TE0, TE1, TE2, and TE3. A statistically significant increase was found in splanchnic FTOE between feeding phases 4 and 0 during TE1 (*P* = .046). Dots represent mean values, and error bars represent 95% CIs.

Table 2, Figure 3, and eTable 2 in the Supplement summarize FTOEs over FPs and TEs (eTable 1 in the Supplement summarizes SpO_2_ and splanchnic and cerebral StO_2_). The primary MMRM analyses found no evidence that the association between FP and FTOEs differed across TEs (eTable 3 in the Supplement gives the FTOE estimates from the primary MMRM model), and there was no evidence of an overall association between FTOEs and FP (FP0: mean estimate, 11.64; 95% CI, 9.55-13.73; FP1: mean estimate, 12.02; 95% CI, 9.92-14.11; FP2: mean estimate, 12.77; 95% CI, 10.68-14.87; FP3: mean estimate, 12.54; 95% CI, 10.45-14.64; FP4: mean estimate, 12.98; 95% CI, 10.89-15.08; *P* = .16 for the FP association). However, in the exploratory set of analyses applied to each transfusion epoch separately, a statistically significant difference was found between FP0 vs FP4 during TE1 (mean [SD] FTOEs during TE1, 10.55 [5.5] for FP0 vs 13.21 [5.96] for FP4; *P* = .046). No feeding-associated changes were found in TE2 and TE3.

### Regional Cerebral Tissue Oxygenation

The primary MMRM analyses of FTOEc found no evidence that the association between FP and FTOEc differed across TEs (eTable 4 in the Supplement gives the FTOEc estimates from this model), and there was no evidence of an overall association between FTOEc and FP (FP0: mean estimate, 22.83; 95% CI, 20.99-24.68; FP1: mean estimate, 23.11; 95% CI, 21.26 -24.96; FP2: mean estimate, 23.05; 95% CI, 21.20-24.90; FP3: mean estimate, 22.39; 95% CI, 20.54-24.24; FP4: mean estimate, 22.37; 95% CI, 20.52-24.21; *P* = .22 for the FP association). Similarly, in the exploratory set of analyses applied to each TE separately, no significant differences were found between FP0 vs FP1 to 4 in any of the transfusion epochs (Table 2, Figure 3, and eTable 2 in the Supplement).

## Discussion

In this study, we rigorously evaluated FTOE through concurrent monitoring of StO_2_ using NIRS and SpO_2_ using a pulse oximeter during 32 hours. Our primary analysis found limited evidence that feeding was associated with a reduction in efficiency of splanchnic oxygen utilization; however, further exploratory analyses of feeding-related changes during different posttransfusion epochs found statistically significant worsening of FTOEs after feeding during the first 8 hours after transfusion (suggesting an increase in oxygen consumption or a decrease in oxygen delivery or both). We found no evidence of an association between feeding and FTOEc, indicating that cerebral tissue oxygenation was protected during feeding in the anemia and posttransfusion periods.

A postprandial increase in splanchnic tissue oxygenation has been demonstrated to occur^28,43^ because of mesenteric vasodilatation, mediated by the enteric nervous system and endothelial-derived substance P.^44^ Lack of increase^15^ or even worsening^16,17^ of postprandial splanchnic oxygenation has been demonstrated in anemia and is proposed to be associated with impairment of reflexes during low oxygenation states.^16^ Our findings demonstrating no evidence of improvement in postprandial FTOEs during anemia (TE0) are in alignment with the these findings.

In our exploratory analysis, there was evidence of an increase in postprandial FTOEs in the first 8 hours after completion of transfusion (TE1), which would indicate a decrease in oxygen utilization efficiency. Given that oxygen consumption is likely to be higher in the postprandial status because of digestive activity, we hypothesize that an increase in FTOEs would reflect the inability to meet the demand owing to inadequate oxygen delivery. The exact reason for this paradoxical inadequate oxygen delivery after transfusion is unknown but could be associated with storage-related changes in red blood cells. This hypothesis is supported by adult, animal, and in vitro studies.^15,21,22,45,46,47^ Marik et al^45^ found that splanchnic oxygen availability is reduced in adults with sepsis after PRBCT with blood stored for more than 15 days and postulated that poorly deformable transfused red blood cells cause microcirculatory occlusion, leading to tissue ischemia. Simchon et al^46^ found that red blood cell transfusion in rats resulted in preferential trapping of red blood cells with reduced deformability in splanchnic circulation. Nair et al^21^ found that red blood cell transfusion with blood stored for 7 days in enterally fed preterm lambs promoted mesenteric vasoconstriction and impaired vasorelaxation by reducing mesenteric arterial endothelial nitric oxide synthase. With the use of Doppler ultrasonography in preterm infants, PRBCT was associated with a decrease in celiac artery flow^47^ and failure of postprandial increase in superior mesenteric artery flow.^15^ Marin et al^22^ found that after transfusions postprandial splanchnic tissue oxygen levels decreased significantly in preterm infants fed during transfusion compared with infants not fed during transfusion.

Although red blood cells are stored under controlled conditions before transfusion, numerous changes can occur (referred to as the *storage lesion*) that may result in reduced oxygen delivery.^48^ In vitro studies have found that storage lesion results in a lack of red blood cell–induced vasodilatation in hypoxic tissues because of the rapid decrease in red blood cell *S*-nitrosohemoglobin,^49^ impaired vasorelaxation attributable to reduced mesenteric arterial endothelial nitric oxide synthase,^21^ and reduced oxygen off-loading and red blood cell deformability attributable to a decrease in the 2,3-diphosphoglycerate level.^50^ The median age of transfused red blood cells in our study was 9 days (IQR, 4-23 days). All red blood cells were leukodepleted, tested negative for cytomegalovirus, and were stored in SAG-M additive solution (sodium chloride, 8.77 g/L; adenine, 0.169 g/L; glucose, 9 g/L; and mannitol, 5.25 g/L). To our knowledge, no prospective studies have used NIRS technology to examine the correlation between the age of red blood cells given to preterm infants and splanchnic tissue oxygenation.

Our finding of worsening postprandial FTOEs during the first 8 hours after transfusion (TE1) that subsequently stabilized (TE2 and TE3) may be attributable to the recovery of a storage red blood cell lesion resulting in improved posttransfusion oxygen delivery. Red blood cell deformability and oxygen unloading recover with time after transfusion. Heaton et al^51^ found that in adults receiving a transfusion, 2,3-diphosphoglycerate levels reached 50% within 7 hours and 95% by 72 hours after transfusion. Marik et al^45^ found similar results, demonstrating that posttransfusion splanchnic ischemia lasted for at least 6 hours after a PRBCT in adults with sepsis. Using NIRS, Marin et al^22^ found that in preterm infants fed during transfusion, the initial decrease in postprandial splanchnic bed oxygen levels returned to the hyperemic response after the fifth feeding after transfusion.

The splanchnic StO_2_ values in our study were higher and corresponding FTOE values lower compared with other published reports.^32,43,52,53,54^ Absolute readings of splanchnic oxygenation are known to vary widely because of peristalsis, stool interference, different monitors, sensors, site of placement of sensors, weight, gestational age, and postnatal age.^35,38,55,56,57,58,59,60,61^ Given that there are no normative values for splanchnic StO_2_, relative changes of tissue oxygenation trends over time are more meaningful compared with absolute values.^59^

The absence of feeding-associated changes in FTOEc indicates that cerebral tissue oxygenation is protected after feedings during anemia and posttransfusion periods. The resilience of cerebral oxygen kinetics, unlike splanchnic oxygen kinetics, underscores the role of cerebral autoregulation.^17,62^

### Strengths and Limitations

This study has strengths. Several studies have examined the association of feeding alone^43,54^ or transfusion alone^31,52,53,63^ with splanchnic or cerebral tissue oxygenation. The strength of this study was the comprehensive investigation of the association of feeding in the context of anemia and transfusion with both splanchnic and cerebral tissue oxygenation. Unlike other researchers,^22,63^ we measured FTOE, which gives a better understanding of oxygen consumption–delivery coupling, rather than only the StO_2_ levels. Rigorous methods and incorporation of high-volume data points in the analysis add to the strength. There is no consensus in the literature regarding transfusion threshold in neonates, and NIRS studies vary in their transfusion threshold.^22,52,53,63,64^ Because of the lack of formal protocol regarding PRBCT, our approach could have been to perform a case-control study with a matched control of infants with similar hemoglobin levels not receiving transfusion. This approach was, however, considered not feasible or rational. Our study was designed so that each infant acted as their control (intraindividual comparison) before vs after PRBCT. Thus, adding an independent control group (without PRBCT) would allow for an interindividual comparison that would have to be corrected for multiple confounders (eg, gestational age, hemoglobin level, sex, cardiorespiratory status, and feeding status) and require a large sample size that would be unfeasible for this study to be completed at a single site in a reasonable time. Moreover, published studies^64,65^ have found poor correlation (correlation coefficient, <0.5) between pretransfusion hemoglobin levels and tissue oxygenation.

This study has limitations. Although a statistically significant change in postprandial FTOEs was found in an exploratory analysis, its biological significance remains uncertain. Only hemodynamically stable infants were included because of concerns that hemodynamic instability may confound splanchnic perfusion independent of feeding. These exclusion criteria may explain why none of the infants studied developed TANEC. The median pretransfusion hemoglobin level in our cohort was 9.3 g/dL (IQR, 8.6-10.3 g/dL). The absence of gut compromise may also indicate that at the transfusion threshold, an increase in FTOEs was perhaps not biologically significant. In addition, the small sample size limits the generalizability of our findings. Moreover, we cannot exclude bias because of the selective enrollment of infants who received PBRCT during office hours. The study involved stable, premature infants with a median postmenstrual age of 34 weeks (IQR, 32.9-35.0 weeks), and as such, caution must be exercised in interpreting results of the study in more premature infants with cardiorespiratory instability. Because of the observational nature of the study, an association rather than the cause-effect relationship can be ascertained between feeding and splanchnic perfusion.

## Conclusions

Our study explored a mechanistic basis underpinning the clinical reluctance to feeding preterm infants in the context of transfusion. We noted the worsening of postprandial splanchnic oxygenation in the first 8 hours after completion of PRBCT and the stability of postprandial cerebral oxygenation in infants with anemia and after PRBCT. Our results warrant further investigation and support the need for adequately powered randomized clinical trials to evaluate the role of feeding in TANEC.

## Notes

Supplement. **eTable 1.** Splanchnic and Cerebral Oxygen Kinetics in Association With Feeding and Transfusion (N = 24) **eTable 2.** Splanchnic and Cerebral FTOE Estimates From Exploratory Analysis **eTable 3.** Splanchnic FTOE Estimates From Primary MMRM Model (N=24) **eTable 4.** Cerebral FTOE Estimates From Primary MMRM Model (N=24)
