# Supplementary material for: Association of Bolus Feeding With Splanchnic and Cerebral Oxygen Utilization Efficiency Among Premature Infants With Anemia and After Blood Transfusion
Source: JAMA Netw Open. 2020 Feb 28;3(2):e200149. doi: 10.1001/jamanetworkopen.2020.0149 (PMC7049081; doi:10.1001/jamanetworkopen.2020.0149)
Supplement: Supplement. — eTable 1. Splanchnic and Cerebral Oxygen Kinetics in Association With Feeding and Transfusion (N = 24) eTable 2. Splanchnic and Cerebral FTOE Estimates From Exploratory Analysis eTable 3. Splanchnic FTOE Estimates From Primary MMRM Model (N=24) eTable 4. Cerebral FTOE Estimates From Primary MMRM Model (N=24) [file jamanetwopen-3-e200149-s001.pdf]

Supplementary Online Content

Balegar V KK, Jayawardhana M, Martin AJ, de Chazal P, Nanan RKH. Association of bolus feeding with splanchnic and cerebral oxygen utilization efficiency among premature infants with anemia and after blood transfusion. *JAMA Netw Open*. 2020;3(2): e200149. doi:10.1001/jamanetworkopen.2020.0149

eTable 1. Splanchnic and Cerebral Oxygen Kinetics in Association With Feeding and Transfusion (N=24)

eTable 2. Splanchnic and Cerebral FTOE Estimates From Exploratory Analysis

e Table 3: Splanchnic FTOE Estimates From Primary MMRM Model (N=24)

e Table 4: Cerebral FTOE Estimates From Primary MMRM Model (N=24)

This supplementary material has been provided by the authors to give readers additional information about their work.

eTable 1. Splanchnic and Cerebral Oxygen Kinetics in Association With Feeding and Transfusion (N=24)

Transfusion epochs	Feeding phases	SpO_2 Mean (SD), %	Splanchnic StO_2, Mean (SD), %	Cerebral StO_2, Mean (SD), %
0	0	96.7 (2.8)	84.8 (6.7)	71.1 (5.6)
	1	96.2 (2.6)	84.6 (6.9)	70.3 (6.0)
	2	96.7 (2.1)	83.8 (6.8)	71.0 (6.2)
	3	96.4 (2.8)	83.7 (6.8)	71.8 (6.0)
	4	97.2 (1.9)	83.7 (7.1)	72.1 (5.3)
1	0	96.1 (1.9)	85.7 (6.1)	76.3 (3.9)
	1	94.9 (3.4)	83.4 (5.7)	74.8 (4.8)
	2	96.0 (1.8)	83.8 (5.6)	75.9 (4.2)
	3	96.3 (1.8)	83.7 (5.3)	76.6 (3.7)
	4	96.4 (1.8)	83.4 (5.4)	76.9 (3.7)
2	0	96.0 (2.2)	85.1 (5.8)	75.2 (6.7)
	1	95.3 (3.1)	83.6 (4.8)	75.0 (5.7)
	2	96.4 (2.2)	84.1 (5.1)	75.3 (4.8)
	3	96.3 (2.4)	84.9 (5.8)	75.9 (5.2)
	4	95.8 (2.5)	84.4 (5.9)	75.6 (5.1)
3	0	96.1 (2.3)	84.0 (7.5)	74.2 (6.2)
	1	95.8 (2.8)	83.5 (6.8)	73.4 (6.2)
	2	96.1 (2.5)	83.8 (7.8)	74.1 (6.0)
	3	96.2 (2.3)	84.3 (8.3)	74.6 (6.4)
	4	95.7 (2.8)	83.4 (7.5)	74.3 (6.8)

SpO_2= pulse oximeter oxygen saturation; StO_2 = tissue oxygen saturation;

© 2020 Balegar V KK et al. *JAMA Network Open.*

eTable 2. Splanchnic and cerebral FTOE estimates from exploratory analysis

TE	FP	Splanchnic FTOE		Cerebral FTOE	
		[N] Mean (SD), %	95% CI, %	[N] Mean (SD), %	(95% CI), %
0	0	[24] 12.06 (7.37)	8.95 - 15.18	[24] 26.23 (5.43)	23.94 - 28.52
	1	[24] 11.68 (7.54)	8.49 - 14.86	[24] 26.67 (5.57)	24.32 - 29.03
	2	[24] 13.01 (6.91)	10.10 - 15.93	[24] 26.41 (5.62)	24.04 - 28.79
	3	[24] 12.92 (6.70)	10.09 - 15.75	[24] 25.40 (5.84)	22.94 - 27.87
	4	[24] 13.59 (7.12)	10.59 - 16.60	[24] 25.58 (5.11)	23.42 - 27.74
1	0	[22] 10.55 (5.50)	8.11 - 12.99	[22] 20.45 (3.04)	19.11 - 21.80
	1	[22] 11.73 (5.18)	9.43 - 14.02	[22] 20.97 (4.14)	19.14 - 22.81
	2	[22] 12.57 (5.70)	10.05 - 15.10	[22] 20.80 (3.74)	19.14 - 22.46
	3	[22] 12.80 (5.26)	10.47 - 15.13	[22] 20.27 (3.31)	18.80 - 21.74
	4	[22] 13.21 (5.96)	10.57 - 15.85	[22] 20.05 (3.50)	18.50 - 21.60
2	0	[23] 11.16 (5.46)	8.80 - 13.52	[23] 21.46 (6.49)	18.65 - 24.27
	1	[22] 12.17 (4.24)	10.29 - 14.04	[22] 21.49 (5.05)	19.26 - 23.73
	2	[22] 12.63 (4.54)	10.62 - 14.64	[22] 21.96 (4.50)	19.97 - 23.96
	3	[22] 11.87 (5.11)	9.61 - 14.14	[22] 21.41 (4.93)	19.23 - 23.59
	4	[22] 11.98 (4.94)	9.79 - 14.17	[22] 21.35 (4.63)	19.30 - 23.41
3	0	[21] 12.79 (7.06)	9.58 - 16.01	[21] 23.00 (5.78)	20.37 - 25.63
	1	[21] 12.55 (6.95)	9.38 - 15.72	[21] 23.46 (6.00)	20.73 - 26.19
	2	[21] 12.85 (7.89)	9.26 - 16.44	[21] 23.22 (5.92)	20.52 - 25.91
	3	[21] 12.53 (8.56)	8.63 - 16.43	[21] 22.69 (6.00)	19.96 - 25.42
	4	[21] 13.09 (7.19)	9.82 - 16.36	[21] 22.69 (5.69)	20.10 - 25.28

TE = Transfusion Epoch; FP = Feeding Phase; FTOE = Fractional tissue oxygen extraction;
CI – Confidence Intervals

© 2020 Balegar V KK et al. *JAMA Network Open.*

e Table 3: Splanchnic FTOE estimates from primary MMRM model (N=24)

	TE0	TE1	TE2	TE3
FP0	12.06 (9.54 to 14.59)	10.47 (7.89 to 13.05)	11.00 (8.45 to 13.56)	13.04 (10.43 to 15.65)
FP1	11.68 (9.15 to 14.21)	11.65 (9.07 to 14.24)	12.01 (9.43 to 14.59)	12.80 (10.19 to 15.41)
FP2	13.01 (10.48 to 15.54)	12.50 (9.92 to 15.08)	12.47 (9.89 to 15.05)	13.10 (10.49 to 15.71)
FP3	12.92 (10.40 to 15.45)	12.73 (10.15 to 15.31)	11.72 (9.13 to 14.30)	12.78 (10.17 to 15.39)
FP4	13.59 (11.06 to 16.12)	13.14 (10.56 to 15.72)	11.82 (9.24 to 14.40)	13.34 (10.73 to 15.95)

P = 0.97 for the FP-by-TE interaction term.

Values represent mean (95% CI), % for splanchnic FTOE
MMRM = Mixed model for repeated measures
TE = Transfusion Epoch; FP = Feeding Phase; FTOE = Fractional tissue oxygen extraction;
CI – Confidence Intervals

© 2020 Balegar V KK et al. *JAMA Network Open.*

e Table 4: Cerebral FTOE estimates from primary MMRM model (N=24)

	TE0	TE1	TE2	TE3
FP0	26.23 (24.14 to 28.31)	20.48 (18.36 to 22.59)	21.71 (19.61 to 23.81)	22.91 (20.77 to 25.04)
FP1	26.67 (24.59 to 28.76)	21.00 (18.88 to 23.11)	21.37 (19.26 to 23.49)	23.37 (21.24 to 25.50)
FP2	26.41 (24.33 to 28.50)	20.82 (18.71 to 22.94)	21.84 (19.72 to 23.96)	23.12 (20.99 to 25.26)
FP3	25.40 (23.32 to 27.49)	20.29 (18.18 to 22.41)	21.29 (19.17 to 23.40)	22.60 (20.47 to 24.73)
FP4	25.58 (23.49 to 27.66)	20.07 (17.96 to 22.19)	21.23 (19.12 to 23.35)	22.60 (20.46 to 24.73)

$P > 0.99$ for the FP-by-TE interaction term.

Values represent mean (95% CI), % for cerebral FTOE
MMRM = Mixed model for repeated measures
TE = Transfusion Epoch; FP = Feeding Phase; FTOE = Fractional tissue oxygen extraction;
CI – Confidence Intervals

© 2020 Balegar V KK et al. *JAMA Network Open.*
